# Dual-Functional Tunable Metasurface for Meta-Axicon with a Variable Depth of Focus and Continuous-Zoom Metalens

**DOI:** 10.3390/nano13182530

**Published:** 2023-09-10

**Authors:** Chang Wang, Yan Sun, Zeqing Yu, Xinyu Liu, Bingliang Chen, Yang Zhang, Zhenrong Zheng

**Affiliations:** 1 College of Optical Science and Engineering, Zhejiang University, Hangzhou 310027, China; changwang_optics@zju.edu.cn (C.W.);; 2 Intelligent Optics & Photonics Research Center, Jiaxing Research Institute, Zhejiang University, Jiaxing 314000, China

**Keywords:** optical metasurface, tunability, multifunctionality, axicon, Bessel beam, zoom metalens

## Abstract

Optical metasurfaces have been widely investigated for their versatile ability to manipulate wavefront and miniaturize traditional optical components into ultrathin planar devices. The integration of metasurfaces with multifunctionality and tunability has fundamentally transformed optics with unprecedented control over light propagation and manipulation. This study introduces a pioneering framework for the development of tunable metasurfaces with multifunctionality, and an example of a tunable metasurface of dual functionalities is proposed and numerically verified as one of the tunable meta-axicon for generating Bessel beams with a variable depth of focus (DOF) and a continuous-zoom metalens. Specifically, this design achieves dual-functional phase modulation by helicity-multiplexing from the combination of the geometric phase as well as the propagation phase and realizes tunability for both functionalities through rotational actuation between double metasurface layers. As a result, dual functionalities with continuous tunability of the proposed TiO_2_ metasurface are enabled independently for the left and right circularly polarized (LCP and RCP) incidences at 532 nm. Specifically, LCP light triggers the metasurface to function as a tunable axicon, generating non-diffracting Bessel beams with variable numerical apertures (NA) and DOFs. Conversely, the RCP incidence induces it to operate as a continuous-zoom metalens and generates variable spherical wavefront focusing on diverse focal lengths. This study not only initially implements the design of tunable meta-axicon, but also achieves the integration of such a tunable meta-axicon and continuous-zoom metalens within a single metasurface configuration. The proposed device could find potential applications in biological imaging, microscopic measurement, laser fabrication, optical manipulation, multi-plane imaging, depth estimation, optical data storage, etc.

## 1. Introduction

Metasurfaces, arrays of subwavelength-spaced artificial nanostructures, have exhibited extraordinary capabilities of locally manipulating the phase, amplitude, and polarization of incident electromagnetic waves. Leveraging their adaptability and efficacy in wavefront shaping, metasurfaces hold the promise of downsizing optical components while enhancing their multifunctionality with high-quality performances and have drawn significant attention across diverse applications such as anomalous refraction and reflection [1,2,3], metalens [4,5,6,7,8,9,10], generation of special light beams like Bessel beams and vortex beams [11,12,13,14,15], meta-hologram [16,17,18], wave plates [19,20], etc.

To date, metasurfaces have evolved from early static and single-function designs that limit their versatility and practicality to multifunctional multiplexing and tunable metasurfaces capable of dynamically modulating light through external manipulation. For one thing, making use of more degrees of design freedom is the fundamental way to develop multifunctional metasurfaces, including strategies of multiplexing parameters such as polarization [21,22,23], wavelength [24,25], or angle [26,27] of incidence. Diverse strategies are explored to develop multifunctionality, such as segmented and interleaved metasurfaces [28,29,30] with functional crosstalk issues and non-interleaved schemes [17,25] that efficiently multiplex independent channels, metasurfaces with non-local supercells [31,32,33,34,35,36] improving wide-angle functionality, multi-layered metasurfaces [37,38,39,40,41,42,43] providing more manipulation degrees, and so on. In addition, the pursuit of dynamic tunability is another primary aim, often achieved through external stimuli. Active materials including liquid crystals (LCs), transparent conducting oxides (TCOs), indium tin oxide (ITO) exhibiting exotic performances at the epsilon-near-zero (ENZ) wavelengths, phase change materials (PCMs), and two-dimensional materials (2-DMs) can be fine-tuned using electrical, thermal, or optical stimuli [44,45], as well as by altering the surrounding medium [46,47,48] of nanostructures. For example, varifocal metalenses by the modification of the local refractive index surrounding the infiltration of different LCs [49]; a reconfigurable metalens that is permeated with a nematic liquid crystal (NLC) and a gold nanoparticle solution and can be tuned by the thermoplasmonic-induced changes of the NLC solution associated with the nematic–isotropic phase transition [50]; two-photon direct laser writing (TP-DLW) achieving hyper-resolution due to extraordinary collimation of the writing laser light enabled by ENZ metamaterials [51]; and so on. Additionally, tunability can be achieved through shifts in material composition via chemical or electrochemical reactions [52,53,54], structural reconfiguration through micro-electro-mechanical systems (MEMS) [54,55] or flexible substrates [56,57], as a tunable visible color-changing metasurface that exploits Fano resonances and could be applied in colorimetric sensing and readily adapted for scalable fabrication has been proposed [58], and researchers also have implemented a locally disordered metamaterial that utilizes water waveguiding dominated by Fano-type interference and Fabry–Pérot resonance and achieves directing and trapping water waves [59]. Moreover, the mutual motion of multilayered metasurfaces by mechanical actuation like Moiré metasurfaces [43] and Alvarez metasurfaces [40,41]. Amid the rapid development in the above research fields, metasurfaces with both multifunctionality and tunability are foreseen and achieved by using strategies like LCs [60], nanoelectrodes [61], external polarizers or retarders [62], etc.

**Figure 1:**
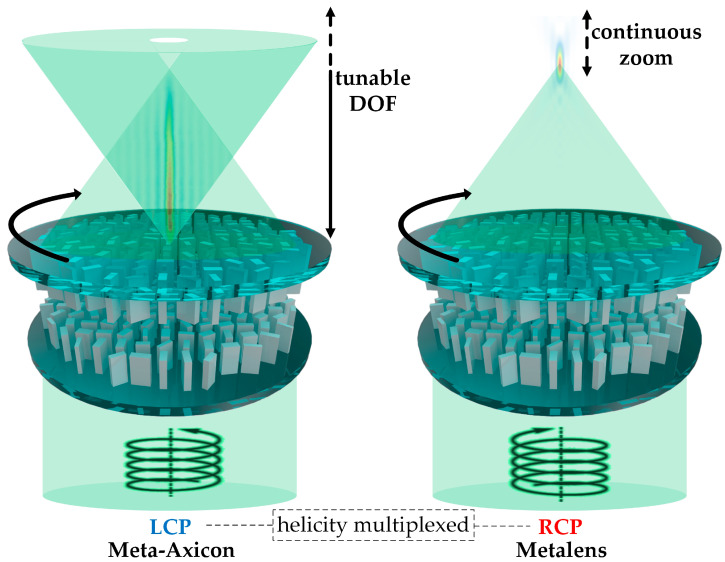
Schematic representation of the DFTM. The nanostructures on both metasurface layers are arranged face to face with a small gap. For LCP incidence, the metasurface functions as a tunable axicon that generates Bessel beams with adjustable DOFs, while for RCP incidence, it operates as a continuous-zoom metalens with changeable focal length.

In this study, a dual-functional tunable metasurface (DFTM) is proposed, as shown in Figure 1. On the one hand, the DFTM initially implements an ultrathin, tunable metasurface axicon (meta-axicon), producing a good approximation of a zero-order Bessel beam featured by its unique non-diffractive and self-healing characteristics [11,12]. This tunable meta-axicon creates a variable focal line rather than a focal point as classical lenses, generating a tunable DOF or NA that can be extendable up to 0.8. On the other hand, the DFTM also achieves the functionality of a continuous-zoom metalens and integrates it with the tunable meta-axicon via helicity-multiplexing, effectively avoiding crosstalk between both functions. As for the working principle, the design consists of a bilayer of cascaded metasurfaces with face-to-face anisotropic titanium dioxide (TiO_2_) nanostructures on two quartz substrates and the dual functionality is achieved by imparting each layer with distinct phase distributions for incidences of opposite helicities, combining geometric and propagation phases [63]. Consequently, the DFTM works as a tunable meta-axicon for LCP incidence and a continuous-zoom metalens for RCP incidence. Furthermore, the tunability is actualized by the mutual rotation of both layers, giving rise to two differently changing phase profiles for opposite helicities. The characteristics of the DFTM are theoretically investigated and numerically validated using the finite difference time domain (FDTD) method. The results illustrate that the DOF or NA of the Bessel beam for LCP incidence and the focal length of the zoom metalens for RCP incidence can be tuned continuously through rotating actuation of both metasurface layers, and the varying ranges for the DOF of the meta-axicon and zoom range for the metalens can be reconfigurable by predesign. This work presents an inventive framework for the design of metasurfaces with both tunability and multifunctionality, and the DFTM is envisioned to find itself important applications in laser fabrication, optical manipulation, multi-plane imaging, optical tomography, data storage, optical communications, etc.

## 2. Materials and Methods

### 2.1. Phase Control Principle of DFTM

The operational principle of the DFTM combines geometric phase *ψ* and propagation phase *η* instead of using space division multiplexing strategies that have low efficiency as well as inflexible adjustment [64]. Geometric metasurfaces (GEMs) impart opposite geometric phase distributions for LCP and RCP incidences through the rotation of nanostructures [63]. In contrast, the propagation phase is dependent on the geometric properties of the nanostructures and insensitive to the polarization states of the incident light [65]. For instance, when a circularly polarized beam is incident on a nanostructure of GEMs [5], the transmitted light can be expressed as

(1) $$E_{t}=\frac{t_{L}+t_{S}}{2}|\sigma \rangle +\frac{t_{L}-t_{S}}{2}\exp(i2\sigma \theta )|-\sigma \rangle ,$$

where the spin-charge *σ* = 1 and *σ* = −1 represent LCP and RCP, respectively, and $|\sigma \rangle ={\left[1+i\sigma \right]}^{T}/\sqrt{2}$ denotes the corresponding unit vector; *t_L_* and *t_S_* are the complex transmittance coefficients for longer and shorter optical axes of the nanostructure, *θ* is its rotation angle along the *z*-axis, and 2*θ* is equivalent to the geometric phase *ψ*. An ideal GEM nanostructure works as a half-wave plate, whose *t_L_* should be equal to *t_S_*∙exp(*i*π) = −*t_S_*, making the first complex coefficient of Equation (1), (*t_L_* + *t_S_*)/2, equal to zero. Then, the second complex coefficient of Equation (1), (*t_L_* − *t_S_*)/2, contains the propagation phase for the output with an orthogonal polarization state. Here, each individual nanostructure within the DFTM is presumed to possess a sufficiently high polarization conversion efficiency (PCE), making (*t_L_* + *t_S_*)/2 regarded negligible, and the complex transmittance can be rewritten by the notation of *T*∙exp(*iη*) = (*t*_L_ − *t*_S_)/2 for simplicity. The proposed DFTM consists of two layers, as shown in Figure 1, and for the first layer, Equation (1) could be rewritten as:

(2) $$E_{1t}=T_{1}\exp[i(\eta_{1}+2\sigma \theta_{1})]|-\sigma \rangle ,$$

and the corresponding output phase distribution becomes [40]:

(3) $$\Phi_{1}{}_{\pm }=\eta_{1}\pm 2\theta_{1}=\eta_{1}\pm \psi_{1},$$

where “+” and “−” are used for LCP and RCP incident light, respectively, and Φ_1+_ as well as Φ_1−_ can be chosen arbitrarily. In addition, Equation (3) indicates that the geometric phase and the propagation phase could be expressed as *ψ*_1_ = (Φ_1+_ − Φ_1−_)/2 and *η*_1_ = (Φ_1+_ + Φ_1−_)/2, respectively. Subsequently, the incident electric field on the second layer, *E*_2*i*_, is assumed to exhibit negligible divergence from *E*_1t_ after passing through the gap that is small enough between the two layers. Similarly, the notation *T*_2_∙exp(*iη*_2_) = (*t*′*_L_* − *t*′*_S_*)/2 is used for anisotropic nanostructures on the second layer, and the final transmitted field *E*_2*t*_ can be derived as:

(4) $$E_{2t}=T_{1}T_{2}\exp\{i[(\eta_{1}+\eta_{2})+2\sigma (\theta_{1}-\theta_{2}]\}|\sigma \rangle ,$$

and as a result, the final output phase distribution of the bilayer metasurface should be:

(5) $$\Phi_{\pm }=\left(\eta_{1}+\eta_{2}\right)\pm 2\left(\theta_{1}-\theta_{2}\right)=(\eta_{1}+\eta_{2})\pm (\psi_{1}-\psi_{2})=\Phi_{1}{}_{\pm }+\Phi_{2\mp }.$$

**Figure 2:**
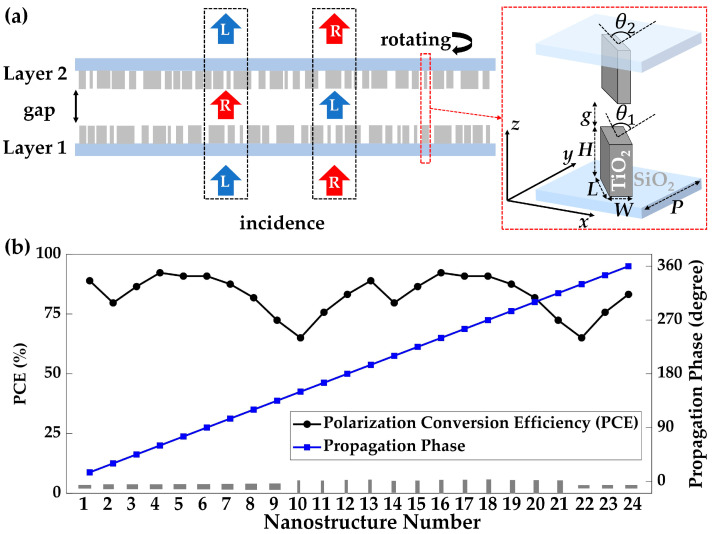
(**a**) Schematic of the working principle of the proposed bilayer metasurface. The TiO_2_ nanostructures on quartz substrates are spaced with a small gap distance *g*, featuring distinct rotational angles for inducing geometric phases and diverse structural parameters for producing propagation phases at various positions on both metasurface layers. A normally incident circularly polarized light interacts initially with the first layer and is converted to an output with opposite helicity. After passing the second layer, the final output light will be in the same polarization state as the incidence, with the overall phase distributions being continuously changed. (**b**) Polarization conversion efficiencies and the propagation phases of the 24-step nanostructures simulated by the commercial package of Lumerical FDTD Solutions. The dimensions of the nanostructures, spanning from 1 to 24, are optimized as follows: lengths (*L*) of 268, 220, 235, 262, 265, 274, 250, 247, 241, 60, 66, 69, 72, 90, 90, 90, 96, 99, 108, 114, 120, 241, 232, and 253 nm; and widths (*W*) of 72, 90, 90, 90, 96, 99, 108, 114, 120, 241, 232, 253, 268, 220, 235, 262, 265, 274, 250, 247, 241, 60, 66, and 69 nm, respectively.

As depicted in Figure 2a, the DFTM imparts distinct phase distributions on orthogonal circularly polarized incidences, LCP and RCP, via both metasurface layers. It transforms the incident plane waves into non-diffracting Bessel beams or spherical wavefront under transmission mode. The nanostructures of the DFTM consist of high-aspect-ratio TiO_2_ nanostructures positioned on quartz substrates, as illustrated in the inset of Figure 2a. TiO_2_ is chosen for this design by virtue of its exceptionally low extinction coefficient (*k*), large refractive index (*n*) and transmittance in the visible range since the high refractive index contrast between TiO_2_ and air/vacuum assures that the energy of the light can be strongly confined within each nanostructures, and the negligible extinction coefficient across the visible spectrum keeps the nanostructures free from Ohmic loss [66]. It is worth noting that while no anisotropic nanostructure could perfectly function as a half-wave plate in line with the assumption of Equation (2), the TiO_2_ nanostructures chosen for this DFTM design exhibit sufficiently high PCE. PCE reflects the fraction of circularly polarized incident light converted into transmitted light with the opposite helicity of the polarization state. The optimized high PCE characteristics displayed in Figure 2b ensure that the geometric phases, realized by rotating nanostructures at various rotation angles, comprehensively cover the 0 to 2π range. Simultaneously, the propagation phases determined by different nanostructure length *L* and width *W* also cover the 0 to 2π span, which is achieved by the 24-step unit cells with an incremental propagation phase of π/12 between neighboring nanostructures.

### 2.2. Design of Meta-Axicon with Variable DOF and NA

Axicons, characterized by their conical lens shape, possess the capability to produce Bessel beams endowed with distinctive attributes such as non-diffraction, self-reconstruction, and optical pulling forces [11,12]. The scalar form of Bessel beams propagating along the *z*-axis can be described in cylindrical coordinates (*r*, *φ*, *z*) [12] by:

(6) $$E(r,\varphi ,z)=E_{\mathrm{amp}}\exp(ik_{z}z)J_{n}(k_{r}r)\exp(\pm in\varphi ),$$

where *E*_amp_ represents the amplitude, while *k_z_* and *k_r_* denote the respective longitudinal and transverse wavevectors. Equation (6) shows that the transverse intensity profiles of Bessel beams are independent of the z coordinate, resulting in their non-diffracting nature.

This can be achieved by an axicon, which symmetrically refracts incident plane waves toward the optical axis of a conical prism to generate a *J*_0_ Bessel beam. The principle of optical phase discontinuities, derived from generalized laws of reflection and refraction [1], can be applied in the design of the proposed meta-axicon. To generate a zeroth-order Bessel beam, a meta-axicon requires a radial phase profile Φ_Axicon_(*r*) [12] of:

(7) $$\Phi_{\mathrm{Axicon}}(r)=-\mathrm{NA}\frac{2\pi }{\lambda }r$$

with a phase gradient of −2πsin*β*/*λ*, where sin*β* is the NA and *β* is the deflecting angle of tan^−1^(*R*/DOF), making NA = sin(tan^−1^(*R*/DOF)) and DOF = *R*/(tan(sin^−1^(NA))). The theoretical NA of a conventional axicon is constrained to a maximum of 0.75 due to the restricted refractive index of silica glasses and total internal reflection within the conical prism. However, the notion of optical phase discontinuities offers a method of designing a meta-axicon with higher NA, and such a phase profile can be imparted to DFTM by the combination of the geometric phase and propagation phase.

The DFTM first proposes a tunable meta-axicon using the following design approach for phase profiles of two layers, generating a varying output phase profile achieved through mutual rotation between both layers as:

(8) $$\Phi_{\mathrm{AX}1}(r,\varphi )=-Ar\varphi -Br$$

and

(9) $$\Phi_{\mathrm{AX}2}(r,\varphi ;\alpha )=Ar(\varphi -\alpha ),$$

where *φ* is the azimuthal angle, *A* and *B* are constant coefficients representing the rate of phase variation of the phase imparted onto the metasurface layers, and *α* is the mutual rotation angle of both metasurface layers. Thus, the output phase profile is theoretically the sum of Φ_AX1_ and Φ_AX2_ as:

(10) $$\Phi_{\mathrm{AX}}(r,\varphi ;\alpha )=-(A\alpha +B)r,$$

which is in the same form as Equation (7), with a varying efficiency of −(*Aα* + *B*) tuned by the rotation angle of *α*. Thus, the NA, as well as the DOF of the proposed meta-axicon, would be expressed as:

(11) $$\mathrm{NA}=\frac{\left(A\alpha +B\right)\lambda }{2\pi }$$

and

(12) $$\mathrm{DOF}=\frac{R}{\tan\left\{\sin^{-1}\left[\frac{\left(A\alpha +B\right)\lambda }{2\pi }\right]\right\}}.$$

In this design, the parameters *A* and *B* should be set to be:

(13) $$A=\frac{2\pi \left(NA_{\max}-NA_{\min}\right)}{\lambda \alpha_{\max}}$$

and

(14) $$B=NA_{\min}\frac{2\pi }{\lambda }.$$

### 2.3. Design of Continuous-Zoom Metalens

Typically, the phase profile of a singlet metalens can either be of a hyperboloidal shape [11], which produces a perfect spherical wavefront, or a quadratic form obtained by considering the first two terms of the Taylor series expansion [37] as:

(15) $$\Phi_{\mathrm{Lens}}(r)=-\frac{2\pi }{\lambda }\left(\sqrt{r^{2}+f^{2}}-f\right)\approx -\frac{\pi r^{2}}{\lambda f}.$$

DFTM introduces a continuous-zoom metalens inspired by varifocal Moiré metalenses [67,68], utilizing the following phase profile design method of quadratic form for both layers, achieving a varying output phase profile through mutual rotation between them:

(16) $$\Phi_{\mathrm{ZL}1}(r,\varphi )=-Cr^{2}\varphi -Dr^{2}$$

and

(17) $$\Phi_{\mathrm{ZL}2}(r,\varphi ;\alpha )=Cr^{2}(\varphi -\alpha ),$$

where *φ* is the azimuthal angle, *C* and *D* are, similarly, the constant coefficients representing the rate of phase variation of the phase imparted onto the metasurface layers, and *α* is the mutual rotation angle of both metasurface layers. Thus, the output phase profile is theoretically the sum of Φ_ZL1_ and Φ_ZL2_ as:

(18) $$\Phi_{\mathrm{ZL}}(r,\varphi ;\alpha )=-\left(C\alpha +D\right)r^{2},$$

which is in the same quadratic form as Equation (15), with a varying coefficient of −(*Cα* + *D*) tuned by the rotation angle of *α*. Then, a tunable focal length proportional to *α* will be generated as:

(19) $$f=\frac{\pi }{(C\alpha +D)\lambda }.$$

In this design, the parameters *C* and *D* should be set to be:

(20) $$C=\frac{\pi (\tan(\sin^{-1}({\mathrm{NA}}_{\max}))-\tan(\sin^{-1}({\mathrm{NA}}_{\min})))}{\lambda R\alpha_{\max}}$$

and

(21) $$D=\frac{\pi }{\lambda f_{\max}}=\frac{\pi \tan(\sin^{-1}({\mathrm{NA}}_{\min}))}{\lambda R}.$$

### 2.4. Phase Profile Design of DFTM

Finally, the phase profiles of both layers, Φ_1±_ and Φ_2±_, can be designed as:

(22) $$\Phi_{1+}(r,\varphi )=-Ar\varphi -Br,$$

(23) $$\Phi_{1-}(r,\varphi )=-Cr^{2}\varphi -Dr^{2},$$

(24) $$\Phi_{2+}(r,\varphi ;\alpha )=Cr^{2}(\varphi -\alpha ),$$

and

(25) $$\Phi_{2-}(r,\varphi ;\alpha )=Ar(\varphi -\alpha ).$$

Thus, according to Equation (5), the propagation phases and geometric phases of both layers can be described by:

(26) $$\eta_{1}(r,\varphi )=\frac{-Ar\varphi -Br-Cr^{2}\varphi -Dr^{2}}{2},$$

(27) $$\eta_{2}(r,\varphi ;\alpha )=\frac{\left(Ar+Cr^{2})(\varphi -\alpha \right)}{2},$$

(28) $$\psi_{1}(r,\varphi )=\frac{-Ar\varphi -Br+Cr^{2}\varphi +Dr^{2}}{2},$$

and

(29) $$\psi_{2}(r,\varphi ;\alpha )=\frac{\left(-Ar+Cr^{2})(\varphi -\alpha \right)}{2}.$$

When an LCP plane wave hits the bottom layer of the DFTM, an output RCP light with a phase profile of Φ_1+_ will be generated, and then it hits the top layer of the DFTM, generating a final output of LCP light with a phase profile of Φ_1+_ + Φ_2−_ that is denoted as Φ_DFTM+_ = −(*Aα* + *B*)*r* and same as Equation (10). Similarly, when the DFTM is hit by an RCP plane wave, the final output would be an RCP light with a converging phase profile of Φ_1−_ + Φ_2+_ that is denoted as Φ_DFTM−_ = −(*Cα* + *D*)*r*^2^—the same as in Equation (18). Hence, the design of the metasurface nanostructures and the phase profile design for both metasurface layers within the DFTM are demonstrated above.

## 3. Results

**Figure 3:**
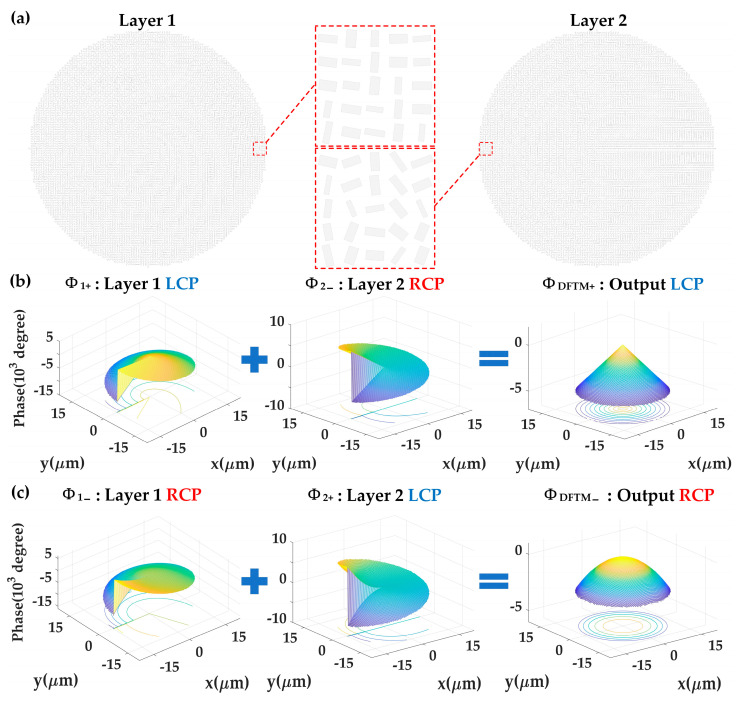
(**a**) Top views of TiO_2_ nanostructures on both metasurface layers within the designed DFTM. The variation in nanostructure arrangements generates diverse combinations of propagation and geometric phases. (**b**) Phase profiles of *φ*_DFTM+ =_ *φ*_1+_ + *φ*_2−_ illustrating the phase manipulation mechanism for meta-axicon functionality under LCP incidence. (**c**) Phase profiles of *φ*_DFTM− =_ *φ*_1−_ + *φ*_2+_ for continuous-zoom metalens functionality under RCP incidence.

The working mechanism of the DFTM nanostructures demonstrated by Equations (22)–(29) can be achieved in two steps: for the first metasurface layer, different phase profiles, Φ_1+_ and Φ_1−_, are separately imparted into the LCP and RCP incidences by opposite geometric phase ±*ψ*_1_ and same propagation phase *η*_1_; for the second metasurface layer, the phase changes of Φ_2+_ and Φ_2−_ are also separately imparted into incidences with opposite helicity by opposite geometric phase ±*ψ*_2_ and same propagation phase *η*_2_, as shown in Figure 3.

The diameter of DFTM is 30 μm, with the NA varying range for both functionalities constrained to [0.5, 0.8], and the mutual rotation angle α within the range [0, π/2] is selected for optimal focusing performance during zooming. The coefficients of A, B, C, and D are determined to be 2.2556 × 10^6^ rad/m, 5.0952 × 10^6^ m^−1^, 1.8947 × 10^11^ rad/m^2^, and 2.2729 × 10^11^ m^−2^, respectively. Parameter optimizations for the proposed nanostructures, as well as subsequent full-wave simulations, were carried out using the FDTD 3D electromagnetic simulator within the commercial Ansys Lumerical 2020 R2 package. Consequently, the nanostructure height *H* is set to be 600 nm, and the nanostructure period *P* is fixed at 300 nm, satisfying the Nyquist sampling criterion (*P* < [*λ*/(1 + NA_max_)]) [69]. For selecting an appropriate value of the gap distance g, half of the Talbot distance that is equal to 2*P*^2^/*λ* would be the optimal distance for superposing phases of two cascaded metasurfaces [70]. However, the Talbot distance in this case is 338 nm, which is not only likely to introduce a non-negligible near-field effect but is also too small for practical axial alignment. Here, 675 nm is selected as the value of the gap distance g, which is twice the Talbot distance and a little larger than the incident wavelength of 532 nm, and this value is not too small for the above concerns but small enough to avoid significant diffraction of the output wavefront generated by the first metasurface layer before it hits the second metasurface layer. In addition, in order to keep the accuracy of the tunability, the relative angular position between both layers before rotating actuation should be located and fixed, and this condition could be satisfied in the situation that the largest DOF appears for LCP incidence or the largest focal length appears for RCP incidence. For the nanostructure simulations, the periodic boundary condition is applied in the *x*-direction and *y*-direction, while the perfect matching layer (PML) boundary condition is applied in the *z*-direction. In the following FDTD simulations of the complete device of DFTM, PML boundary conditions are applied in the *x*-direction, *y*-direction and *z*-direction. The simulated results for both the meta-axicon and continuous-zoom metalens functionalities are discussed in Section 3.1 and Section 3.2 below.

### 3.1. Characteristics of the Meta-Axicon Functionality

**Figure 4:**
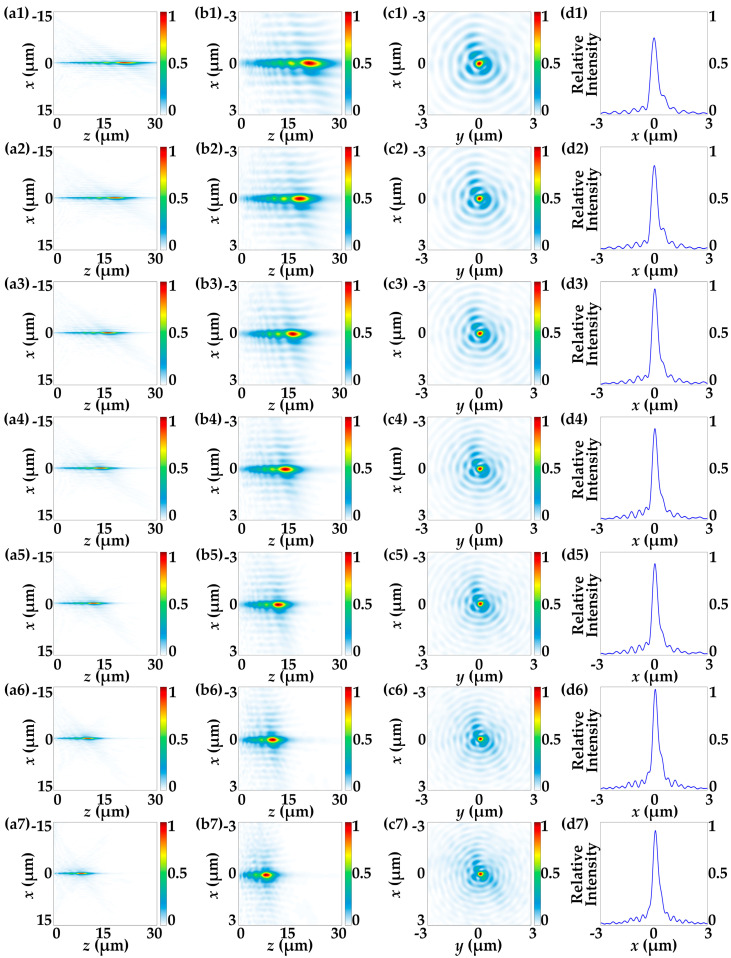
Tunability of the meta-axicon functionality in DFTM under LCP incidence. (**a1**–**a7**) Normalized electric field intensity profiles of Bessel beams generated by the meta-axicon with different DOFs under various rotation angles from 0–90° in the *x*–-*z* plane. (**b1**–**b7**) Zoomed-in results of (**a1**–**a7**) showcasing finer details. (**c1**–**c7**) Corresponding intensity profiles in the *x*–*y* plane provide insights into the lateral distributions of the beams. (**d1**–**d7**) Corresponding intensity profiles along the *x*-axis.

Following the imposition of the corresponding geometric and propagation phases calculated using Equations (26)–(29) on both layers of the DFTM, full-wave simulations under LCP incidence were initially conducted using FDTD to explore the performance of the meta-axicon functionality. Figure 4 illustrates the tunability of the meta-axicon functionality in the DFTM under LCP incidence, showcasing results when the top metasurface layer is rotationally actuated at angles of 0°, 15°, 30°, 45°, 60°, 75° and 90°, respectively. The intensity profiles of the electric fields depicted in Figure 4 validate the effectiveness of the generalization of zero-order Bessel beams, as evidenced by the prominent non-diffracting regions. Notably, the sizes of the Bessel beams exhibit continuous tuning as the angle *α* is altered, signifying that the DOFs and NAs of the LCP-driven meta-axicon are dynamically adjusted along the propagation direction.

**Figure 5:**
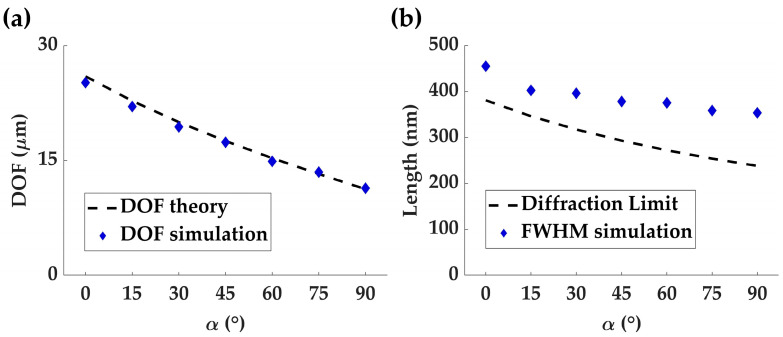
(**a**) The graph showcasing a comparison between the theoretical and measured DOF results for Bessel beams created under LCP incidence while varying the rotation angles at 0°, 15°, 30°, 45°, 60°, 75° and 90°. (**b**) The comparison between the corresponding theoretical and simulated FWHM values for the same rotation angles offers insights into the beam characteristics.

The measured DOFs for *α* values of 0°, 15°, 30°, 45°, 60°, 75° and 90° are 25.15 μm, 21.53 μm, 19.39 μm, 17.37 μm, 14.88 μm, 13.48 μm, and 11.38 μm, respectively. As depicted in Figure 5a, these results closely match the predictions of Equation (12) (dashed line in Figure 5a). Additionally, Figure 4d1–d7 presents the electric field intensity profiles along the *x*-axis, with corresponding full width at half maximum (FWHM) values of 455 nm, 403 nm, 396 nm, 378 nm, 375 nm, 359 nm, and 354 nm for *α* values of 0°, 15°, 30°, 45°, 60°, 75° and 90°, respectively. It is worth noting that the theoretical FWHM value of the zeroth-order Bessel beam *J*_0_ is defined as the FWHM of the center bright spot and can be derived from Equation (6) as 0.38*λ*/NA [12]. However, ideal Bessel beams are not spatially confined, carrying infinite energy, and can only be approximated within a finite region through the superposition of multiple plane waves. Therefore, the simulated FWHM results closely approximate the theoretical values but do not match exactly, as illustrated in Figure 5b.

**Figure 6:**
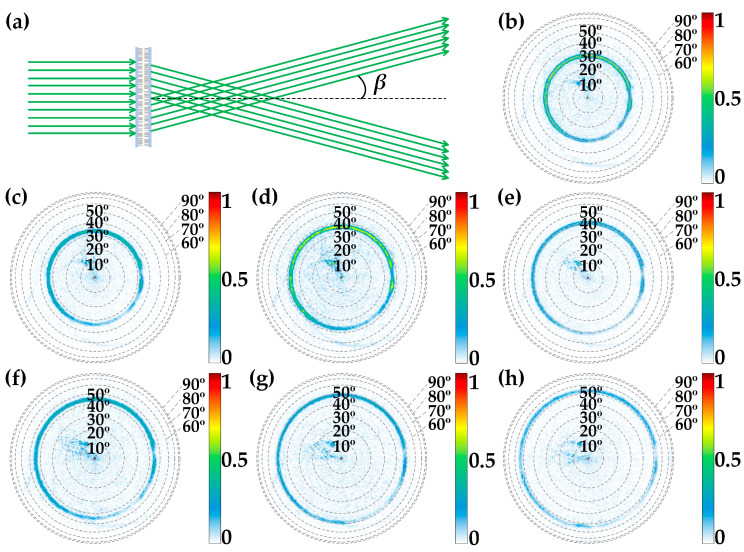
(**a**) Diagram illustrating the far-field ring-shaped beam of the meta-axicon. (**b**–**h**) The FDTD-simulated outcomes of the far-field projection, displayed as images of the far-field intensity on a hemisphere surface with a radius of 1 m, were observed from above. These results correspond to the rotation of the top metasurface layer at angles of 0°, 15°, 30°, 45°, 60°, 75° and 90°, respectively, providing a comprehensive view of the far-field focusing patterns under different rotation conditions.

Furthermore, the far-field propagating characteristics of the meta-axicon should be explored. Unlike Gaussian beams that deteriorate over distance, the beam profile produced by an axicon begins by nearly propagating the properties of a Bessel beam, which maintains a stable intensity distribution as it propagates within the beam overlapping region (DOF) and beyond DOF within the non-diffracting region, axicon could produce a uniform ring-shaped beam [11,12], as depicted in Figure 6a. The theoretical behavior of an axicon predicts the focus of the incident plane wave to form a ring-shaped beam in the far field with a deflection angle of *β* = tan^−1^(*R*/DOF). Figure 6b–h are simulated results obtained by projecting the far-field propagation patterns from a hemisphere surface vertically onto an *x*–*y* plane, and the projected deflection angles for *α* values of 0°, 15°, 30°, 45°, 60°, 75° and 90° are 30°, 33.37°, 36.87°, 40.54°, 44.43°, 48.59° and 53.13°, respectively. Notably, the simulation results in Figure 6b–h agree well with these predicted deflection angles, validating the accuracy of simulated deflection angles of the focusing ring patterns on the far field for the aforementioned *α* values.

### 3.2. Characteristics of the Continuous-Zoom Metalens Functionality

**Figure 7:**
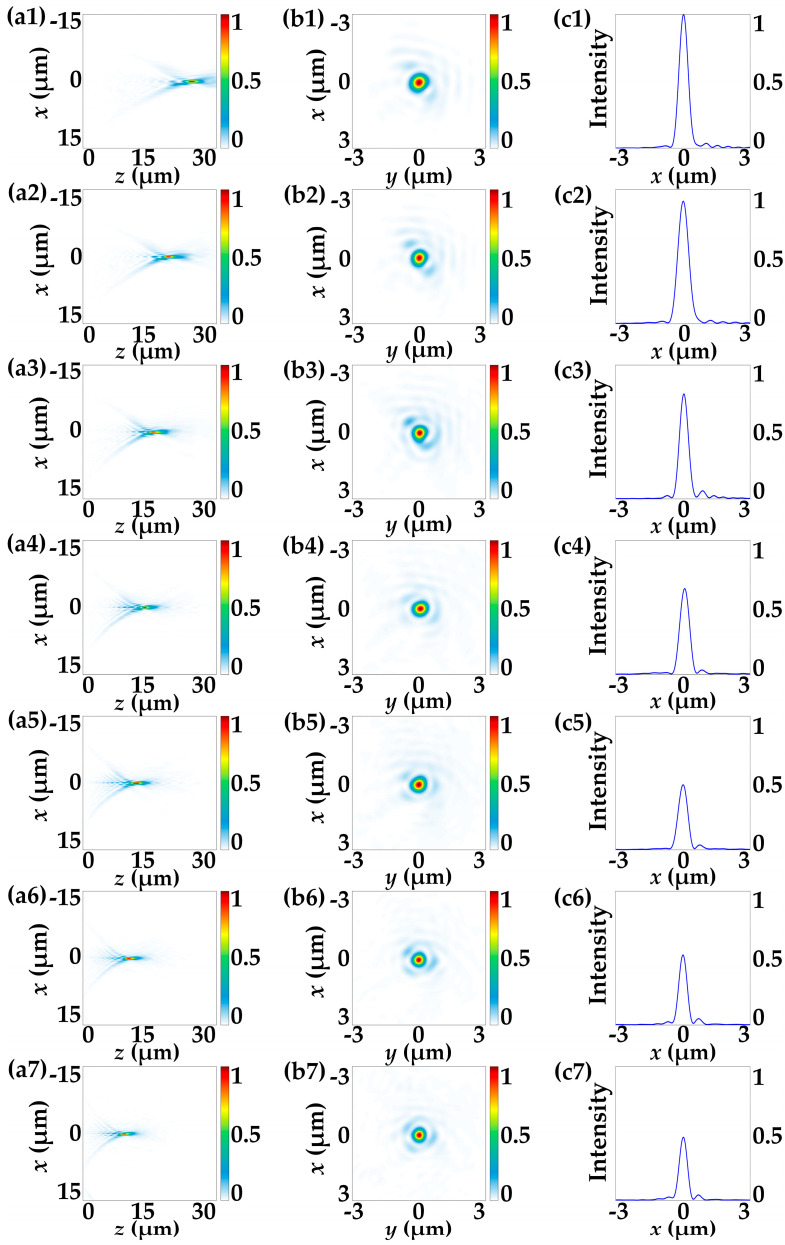
The simulated electronic field intensity distributions of the varifocal performance of the DFTM under RCP incidence. (**a1**–**a7**) Electric field intensity distributions in the *x–z* plane under 532 nm RCP incidence when the top metasurface layer is rotationally actuated by 0°, 15°, 30°, 45°, 60°, 75° and 90°, respectively. (**b1**–**b7**) The corresponding zoom-in intensity profiles of (**a1**–**a7**) reveal the transverse distribution in the *x–y* plane. (**c1**–**c7**) The corresponding relative intensity profiles of (**b1**–**b7**) along the *z*-axis.

Based on the theoretical analyses of Equations (15)–(21), the DFTM works as a varifocal metalens with a single focal spot under the illumination of 532 nm RCP incidences. The designed zoom range spans from 25.98 μm (π/(*Dλ*)) to 11.25 μm (π/[(π*C*/2 *+ D*)*λ*]) with the aperture diameter being of 30 μm, resulting in a large NA that ranges from 0.5 to 0.8. To investigate the continuous-zoom properties of the DFTM, full-wave simulations under RCP incidence by FDTD are then conducted to explore the performances of the continuous-zoom metalens functionality, and varifocal spots by different rotational angles of the top metasurface layer are evidenced by the intensity distributions depicted in Figure 7. Figure 7 illustrates the continuous-zoom metalens functionality in DFTM under RCP incidence when the top metasurface layer is rotationally actuated by 0°, 15°, 30°, 45°, 60°, 75° and 90°, respectively. The intensity profiles of the electric fields shown in Figure 7a1–a7 validate the effectiveness of the varifocal focusing property, and it is evident that the focal lengths, as well as NAs, are continuously tuned along the propagation direction as the angle *α* is changed. This behavior aligns well with the predictions of Equation (19), further confirming the zooming property for the RCP-driven metalens within the DFTM.

**Figure 8:**
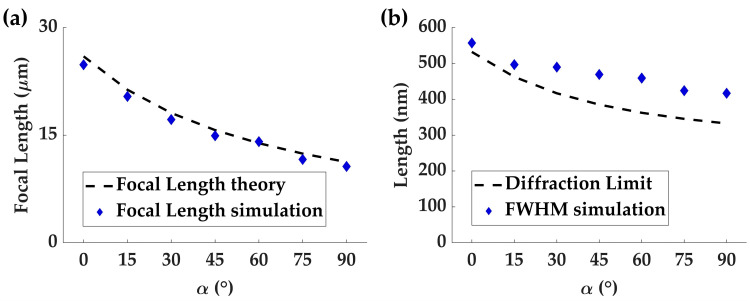
(**a**) The comparison of theoretical and measured focal length results for continuous-zoom metalens functionality under RCP incidence at rotation angles of 0°, 15°, 30°, 45°, 60°, 75° and 90°, respectively. (**b**) The comparison between the simulated FWHM values and the theoretical diffraction limits for the same rotation angles (0°, 15°, 30°, 45°, 60°, 75° and 90°) provides insights into the achievable spot sizes and their proximity to the diffraction limit.

The trends observed in Figure 8a are evident, with an increase in the rotation angle *α* from 0° to 90° (incremented by 15°), leading to a reduction in the focal length of the focal spot formed by the 532 nm RCP incidence. The measured focal lengths for *α* of 0°, 15°, 30°, 45°, 60°, 75° and 90° are 24.81 μm, 19.89 μm, 16.67 μm, 14.91 μm, 13.31 μm, 11.62 μm, and 10.63 μm, respectively. Furthermore, Figure 8b displays the corresponding FWHM values for α of 0°, 15°, 30°, 45°, 60°, 75° and 90°, which are 557 nm, 497 nm, 490 nm, 469 nm, 459 nm, 424 nm, and 416 nm, respectively. These results highlight that the FWHMs describing the spot sizes of these foci are approaching the diffraction limit, which is defined as the working wavelength divided by twice the NA. This indicates that the continuous-zoom metalens functionality within the DFTM exhibits excellent focusing performance, and the variation in focal spot sizes is attributed to their different NAs.

## 4. Conclusions

In conclusion, a dual-functional metasurface combining a function of meta-axicon with tunable DOF and a function of zoom metalens, namely DFTM, is proposed. It is characterized by four aspects: above all; this device initially implements an ultrathin and tunable meta-axicon with adjustable DOF as well as NA; in addition, DFTM innovatively combines the tunable meta-axicon with a continuous-zoon metalens by helicity-multiplexing rather than a segmented or interleaved metasurface design, avoiding functional crosstalk and low operational efficiency; moreover, DFTM utilizes two cascaded metasurfaces layers with distinct phase distributions for incidences with opposite helicities imparted on each layer by the combination of the geometric phase and The propagation phase; finally, by enabling relatively rotating actuation between both layers, the DOF of the meta-axicon functionality for LCP incidence and the zoom range of the continuous-zoom metalens functionality for RCP incidence can be tuned continuously, with both corresponding varying ranges predesigned deliberately. DFTM contributes to the progress of replacing static monofunctional metasurfaces with their counterparts with tunability and multifunctionality, develops the design of tunable meta-axicon for the first time, and initially multiplexes tunable meta-axicon and zoom metalens in one metasurface design. It could be envisioned that this design may find potential applications from biological imaging, microscopic measurement, laser fabrication, and optical manipulation to multi-plane imaging, optical tomography techniques, optical data storage, optical communications, etc.

## Data Availability

The data underlying the results presented in this paper are not publicly available at this time but may be obtained from the authors upon reasonable request.
